# Studies on *In situ* Hydrogel: A Smart Way for Safe and Sustained Ocular Drug Delivery

**DOI:** 10.4103/0975-1483.63144

**Published:** 2010

**Authors:** DH Shastri, LD Patel, RK Parikh

**Affiliations:** *Department of Pharmaceutics, K.B. Institute of Pharmaceutical Education & Research, Sector 23, Gandhinagar, Gujarat-382 023, India*; 1 *Professor and Principal, Faculty of Pharmacy and Research, Wadhwan, Surendranagar-380007, India*; 2 *Department of Pharmaceutics, L.M. College of Pharmacy, Ahmedabad, India*

**Keywords:** Carbopol 940, cromolyn sodium, *in situ* gelation, ophthalmic delivery, Pluronic F 127

## Abstract

The present work describes the formulation development of ophthalmic *in situ* gelling system using thermo-reversible gelling polymer, i.e. Pluronic F 127 (PF127). Because of high concentration (20 to 25%w/v) of this polymer required for *in situ* gelation causes irritation to the eye. So, to reduce this concentration, an attempt was made to combine the PF127 with other polymers like hydroxy propyl methyl cellulose (HPMC) as a viscosity increasing agent or with polymers like carbopol 940, xanthan gum, and sodium alginate (high glucuronic acid content) showing a pH and cation-triggered sol-gel transition, respectively. Different batches were prepared of varying concentrations of these polymers with PF127 using cromolyn sodium 2%w/v in phosphate buffer pH 5.0. The formulations were optimized by the viscosity measurement and *in vitro* gelation study. Selected formulations were evaluated for *in vitro* drug release profile and indicated sustain drug release over a period of 10 h. Effect of sterilization on drug content, pH, clarity, and viscosity were also evaluated. Finally, we concluded that by using this type of combination system, we could reduce not only the concentration of individual polymers but also the side effects without compromising the *in vitro* gelling capacity as well as overall rheology of the system.

## INTRODUCTION

One of the main problems encountered in ophthalmic drug delivery is the rapid and extensive elimination of conventional eye drops from the eye. This process results in extensive drug loss. Consequently, only a small amount (1-6%) actually penetrates the cornea and reaches the intra ocular tissues.[1,2] The reasons for this inefficient drug delivery includes rapid tear turnover, lachrymal drainage, and drug dilution by tears.[3] The higher drainage rate is due to tendency of the eye to maintain its residence volume at 7-10 μl permanently, whereas volumes of topically instilled range from 20 to 50μl. It has been demonstrated in vivo that 90% of the dose was cleared within 2 min for an instilled volume of 50 μl.[4] Consequently, the ocular residence time of conventional solution is limited to few minutes, and the overall absorption of a topically applied drug is limited to 1-10%[5] Consequently, most drugs get systemically absorbed via the nose or gut after draining from eye. This excessive systemic absorption not only reduces the ocular bioavailability, but also may lead to unwanted side effects and toxicity.

To increase ocular bioavailability and duration of the drug action, various ophthalmic vehicles, such as viscous solutions, ointments, gels, or polymeric inserts, have been used.[6] The corneal contact time has been increased to varying degrees by these vehicles, but because of blurred vision (i.e. ointments) or lack of patient compliance (i.e. inserts), they have not been widely accepted.

From the point of view of patient acceptability, a liquid dosage form that can sustain drug release and remain in contact with the cornea of the eye for extended periods of time is ideal. If the precorneal residence time of a drug could be improved from 5 min to say a few hours, then improved local bioavailability, reduced dose concentrations and dosing frequency, and improved patient acceptability may be achieved. Drug delivery systems based on the concept of *in situ* gel formation should provide these properties. Such delivery systems consist of phase transition polymers that are instilled in a liquid form and shift to the gel phase once in the cul-de-sac of the eye. Several polymers, demonstrating phase transition due to changes in their microenvironment, were investigated. Among them are poloxamer 407[7] and tetronics,[8] as well as ethyl (hydroxyethyl) cellulose,[9] whose solution viscosity increases upon increasing the temperature to that of the eye, cellulose acetophthalate (CAP) latex,[10] that coagulates when its native pH of 4.5 is raised by the tear fluid to pH 7.4 and Gelrite^TM^, a polysaccharide which gels in the presence of mono or divalent cations.[11] However, most of these vehicles are characterized by a high polymer concentration (25% poloxamer, 30% CAP) which is not well tolerated by the eye.

The present study demonstrate that a reduction in pluronic concentration, without compromising the *in situ* gelling capabilities as well as overall rheological properties of the delivery system, can be achieved by addition of inert polymer acting as viscosity enhancer like HPMC or polymer that shows *in situ* sol-gel transition via another mechanism with desired rheological properties and ability to deliver the drug in sustained manner.

## MATERIALS AND METHODS

### Materials

PF127 obtained from BASF India Ltd, sodium alginate (Manugel DMB) from M. S. Uni., Baroda, Xanthangum obtained from Glasston Ltd, Bhavnagar, Carbopol 940 from Corel Pharma, Ahmedabad, HPMC (Hydroxy propyl methyl cellulose) obtained from Colorcon India Ltd, PVA (Polyvinyl alcohol) and cromolyn sodium were obtained from Indiana Ophthalmic, Bhavnagar. Other ingredients used were of analytical grade and include citric acid, sodium dihydrogen phosphate, sodium chloride, sodium bicarbonate, benzalkonium chloride, disodium editate, calcium chloride, and sterile distilled water.

### Methods

#### Preparation of In situ gel formulation

First of all the aqueous dispersions of the selected concentrations of Carbomer 940 (0.3%, 0.4%, and 0.5%) respectively for batches (C1, C2, and C3) and Sodium Alginate (0.3, 0.4, 0.5%, 0.7%, 0.9%, and 1.5%) respectively for batches (S1, S2, S3, S4, S5, and S6) and Xanthan gum (0.3%, 0.4%, 0.5%) respectively for batches (X1, X2, and X3) in citro phosphate buffer pH 5.0 were prepared for the control batches and for the further study. Similarly, for preparation of pluronic dispersions, varying concentrations of PF127 (10%, 12%, 14%, 15%, and 16%) respectively for batches (P1, P2, P3, P4, P5) were dispersed in the buffer solution with continuous stirring for 5 min. The partially dissolved pluronic solutions were stored in the refrigerator until the entire polymer was completely dissolved (approximately 24 h). Similarly, the batches for pluronic/carbopol (batches CP1 to CP15) and pluronic/alginate (batches SP1 to SP11) and pluronic/Xanthan gum (batches XP1 to XP5) solutions were prepared by dispersing the pluronic in the desired concentrations of respective polymer solutions. The partially dissolved solutions were then refrigerated until thoroughly mixed (approximately 24 hrs). All the previous sample solutions were adjusted to pH 5.0±0.1 with 0.5 M hydrochloric acid solution and then stored in the refrigerator before evaluation of their properties under non-physiological conditions (pH 5.0 and 25°C).[10] The same procedure was used to prepare the dispersions for characterization under physiological conditions (pH 7.4 and 37 °C); the simulated tear fluid (NaCl 0.67 g, NaHCO_3_ 0.20 g, CaCl_2_*2H_2_O 0.008 g, and distilled deionized water to 100 g), however, was used as a dispersion medium instead. The prepared solutions were adjusted to pH 7.4±0.1 with 0.5 M sodium hydroxide solution and then stored in the refrigerator before evaluation of their properties under physiological conditions. In the prepared batches 2%w/v cromolyn sodium and other adjuvants were incorporated. The detailed procedure for the preparation of *in situ* gel forming system is as follows.

The buffer salts were dissolved in about 70 ml of sterile distilled deionized water. Then polymers were sprinkled and allowed to hydrate overnight. The drug cromolyn sodium was dissolved in another 20 ml of sterile distilled water and required quantities of disodium editate as chelating agent and sodium chloride as osmogen were incorporated. Benzalkonium chloride was then added as a preservative. The drug solution was finally added to polymer solution with constant stirring. The pH of the system was adjusted to 5.0±0.1 with 0.5 M NaOH solution. Final volume was adjusted with distilled water.

All formulations were prepared in citro phosphate buffer pH 5.0 were stored in the refrigerator and the rheological profiles of each solution were measured at 12 rpm for comparative evaluation study using Brook field viscometer at 25°C and at 37°C.

#### Viscosity and in vitro gelling efficiency

The prepared formulations were evaluated for *in vitro* gelling efficiency and viscosity in order to identify the compositions that best suit for the use as *in situ* gelling systems [Table 1]. The *in vitro* gelling efficiency was determined by placing a drop of the system in a test-tube containing 2 ml of simulated tear fluid (STF) freshly prepared and equilibrated at 37 °C. The visual assessment of gel formation was carried out simultaneously the time required for gelation as well as time taken for the formed gel to dissolve was also noted. The viscosity of the systems was measured using Brookfield viscometer (LVT model) at 12 rpm for the purposes of comparative evaluation. The flow behavior of vehicles was determined by various signs obtained by visual inspection. The flow behavior with the “+” sign indicates the vehicle is in the liquid form and is very easy to flow which shows mild gelation after a few minutes and the gel dissolves rapidly. The “++” sign indicates that the vehicle is in the liquid-gel like form and flows less readily, which shows gelation immediate the gel remains for ≤1 h. The flow behavior with the “+++” sign indicates that the sample is in the gel form and is very difficult to flow which also shows immediate gelation and the gel remains for the extended period of time. The flow behavior with the “++++” sign indicates that the vehicle is a strong gel and cannot flow at 25°C and 5.0 pH.

**Table 1:** Viscosity and *in vitro* gelling capacity of prepared formulations

Formulation code	Concentration (%w/v)				Gelling capacity*
CP1	10	0.3	--	--	+
CP2	12	0.3	--	--	+
CP3	14	0.3	--	--	+
CP4	15	0.3	--	--	++
CP5	16	0.3	--	--	++
CP6	10	0.4	--	--	++
CP7	12	0.4	--	--	++
CP8	14	0.4	--	--	++
CP9	15	0.4	--	--	+++
CP10	16	0.4	--	--	++++
CP11	10	0.5	--	--	++
CP12	12	0.5	--	--	++
CP13	14	0.5	--	--	+++
CP14	15	0.5	--	--	++++
CP15	16	0.5	--	--	++++
XP1	10	--	0.5	--	+
XP2	12	--	0.5	--	++
XP3	14	--	0.5	--	++
XP4	15	--	0.5	--	+++
XP5	16	--	0.5	--	++++
SP1	14	--	--	0.3	+
SP2	15	--	--	0.3	+
SP3	14	--	--	0.4	+
SP4	15	--	--	0.4	++
SP5	14	--	--	0.5	+
SP6	15	--	--	0.5	++
SP7	14			0.7	++
SP8	15			0.7	+++
SP9	14	--	--	0.9	++
SP10	15	--	--	0.9	+++
SP11	15			1.5	++++

* +Mild gelation after a few minutes, gel dissolves rapidly

++ Gelation immediate, remains for ≤1 hr

+++ - Gelation immediate remains for extended period. - +++ selected formulations.

#### Rheological study

**Figure 1:**
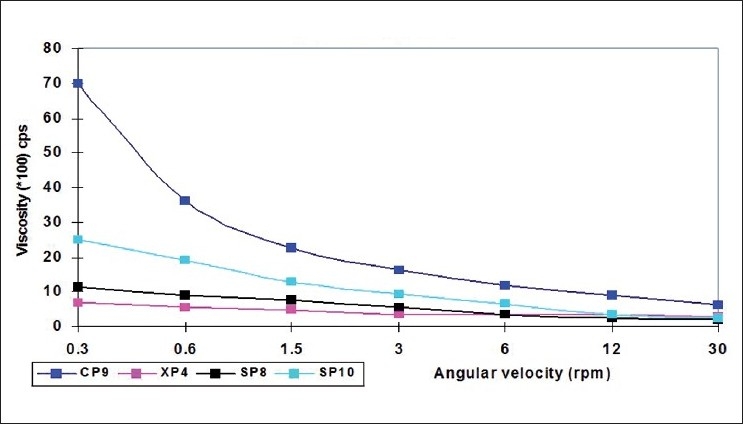
Comparative rheological profile of the selected formulations at pH 5.0 and 25°C

**Figure 2:**
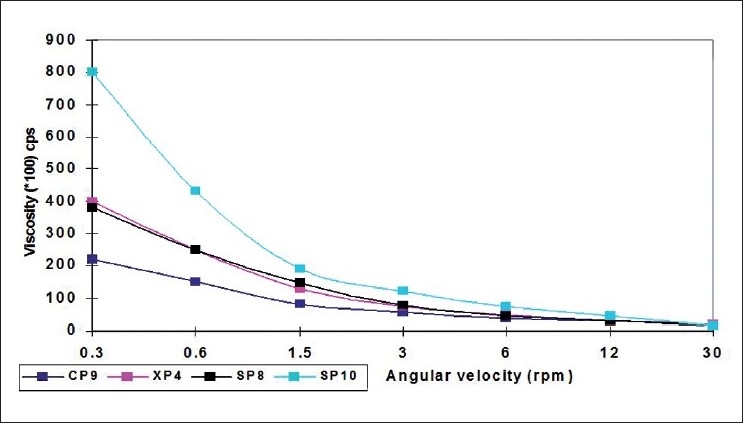
Comparative rheological profile of the selected formulations at pH 7.4 and 37°C

The selected formulations (pH 5.0) at 25°C were poured into the sample adaptor of the Brookfield viscometer (LVT model) and the angular velocity was increased gradually from 0.3 to 30 rpm. The hierarchy of the angular velocity was reversed and average dial reading was considered to calculate the viscosity. Same formulations were again evaluated for viscosity in a similar fashion after adjusting their temperature to 37 °C and pH to 7.4 by adding 0.5M NaOH. The temperature was maintained within ±1.0 °C by a recirculating bath connected to the sample cup of the viscometer. The samples were equilibrated on the plate for 5 min to reach the running temperature before each measurement. The viscosity measured at both the conditions for selected formulations were plotted versus the angular velocity (rpm). Comparative rheological profiles were shown for all three formulations together in Figures 1 and 2. All the measurements were taken in triplicate.

#### In vitro drug release study

**Figure 3:**
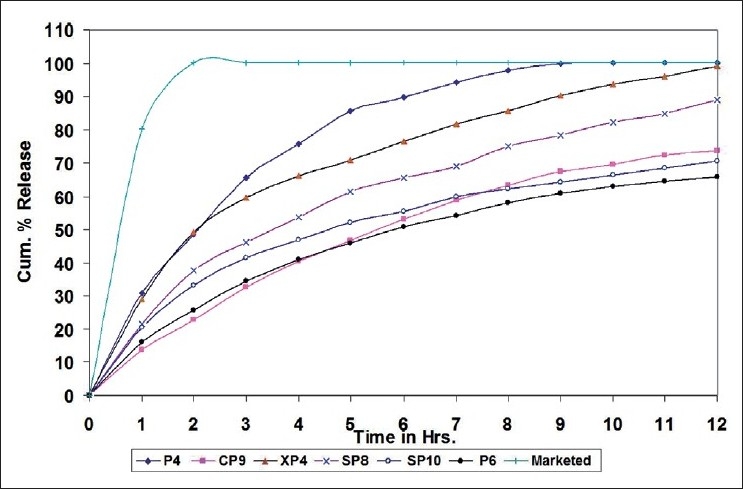
Comparative rheological profile of the selected formulations at pH 7.4 and 37°C

The *in vitro* release pattern of cromolyn sodium was studied using modified dissolution test apparatus.[12,13] Freshly prepared simulated tear fluid (pH 7.4) was used as dissolution medium. Cellophane membrane, previously soaked overnight in the dissolution medium, was tied to one end of a specially designed glass cylinder (open at both ends and of 3 cm diameter). 2 ml of the formulation was accurately pipetted into the cylinder and was suspended in 500 ml of dissolution medium maintained at 37 ±1°C in such a way that the membrane just touched the surface of the receptor medium. The dissolution medium was magnetically stirred at 100 rpm. 10 ml aliquots were withdrawn every hour replacing equal volume of the fresh dissolution medium and were analyzed directly by UV spectrophotometer at 326 nm (Hitachi U-2000). All the selected formulations (CP9, XP4, SP8 and SP10) were evaluated for the drug release pattern and were compared with the release pattern of control batches of PF127 25% (P6) and 15% (P4), carbomer (C2), xanthan gum (X1), sodium alginate (S4 and S5). Also, the drug release profile of the marketed eye drops (Cromal eye drops - Cipla) was compared with the same obtained with selected formulations as shown in Figure 3.

#### Effect of sterilization

The selected formulations were filled in 10 ml capacity amber glass vials, closed with grey butyl rubber closures and sealed with aluminum caps. The vials were subjected to terminal sterilization by autoclaving at 121 °C and 15 psi for 20 min. The formulations were evaluated for drug content, viscosity, clarity, and pH before and after the terminal sterilization.

## RESULTS AND DISCUSSION

### Viscosity and *in vitro* gelling capacity

The two main prerequisites of an in situ gelling system are viscosity and gelling capacity. The formulation should have an optimum viscosity that will allow easy instillation into the eyes and would undergo a rapid sol-gel transition at the body temperature 37 °C and lachrymal pH 7.4. Also, the gel formed *in situ* should preserve its integrity without dissolving or eroding for a sufficient period of time. Table 1 shows gelling efficiency and viscosity of different formulations under study. Formulations coded as CP9, XP4, and SP10 (“_”Underline batches) were selected on the basis of their low viscosity with higher gelling capabilities and on the basis of clarity of the gel formed in the eye.

### Rheological profile

Remarkable increase in the viscosity was observed as the pH of the formulations was increased to 7.4 and temperature to 37 °C. Also, the formulations were found shear thinning, as decrease in viscosity was observed with an increase in angular velocity (pseudoplastic rheology). The administration of ophthalmic preparation should influence the pseudoplastic behavior of tear film as little as possible.[14] The ocular shear rate is very large ranging from 0.03 S^-1^ during inter-blinking periods to 4250-28 550 S^-1^ during blinking. So, viscoelastic fluids with a viscosity that is high under the conditions of low shear rate and low under the conditions of high shear rate are preferred.[13] At pH 5.0 and temperature 25 °C, the formulations were in liquid state and exhibited low viscosity while at pH 7.4 (lachrymal pH) and temperature 37 °C (physiological conditions), transformation of solutions into gel with higher viscosity was observed.

### *In vitro* drug release study

The cumulative percentage release profiles of all selected batches obtained were compared with that of a market formulation [Figure 3]. In case of the marketed eye drops, almost all the drug was released within 60 min. On the other hand, all selected formulation exhibited slow release of the drug. Formulation coded as XP4 showed an initial high burst release of the drug nearly 30%. It may be due to long gelation time taken by xanthan gum. Other formulations exhibited faster gelling and did not show burst release. The combination of polymers of different mechanism of *in situ* gelation with PF127 shows better improvement in *in vitro* drug release profile than that shown by the individual polymers.

The results suggest that combination of this type of polymers of different mechanisms of *in situ* gelation can be utilized as viscosity enhancer together with thermo-reversible gelling polymer like PF127 in an *in situ* gelling system for ophthalmic drug delivery. The *in vitro* release of cromolyn sodium from all the formulations occurred primarily by diffusion. The results clearly showed that the formulations have an ability to retain drugs for more than 10 h period without premature drug release. However, the conditions during *in vitro* drug release experiments were very different from those likely to be encountered in the eye. In the cul-de-sac, the formulations will probably undergo faster dissolution due to the shearing action of eyelid and eyeball movement.

### Effect of sterilization

The autoclaving exerted insignificant effect on the drug content, viscosity, and pH of the formulations [Table 2]. However, haziness was observed in formulations coded as CP9 after autoclaving due to gelation of Pluronic at elevated temperature. But it was found to disappear and the original clarity was regained after overnight storage at ambient conditions.

**Table 2:** Effect of sterilization on selected formulations

Formulation Code	Drug Content %w/v		Viscosity (cps) At 12 rpm		pH	
	Before stern	After stern	Before stern	After stern	Before stern	After stern
CP9	98.50	98.09	887.5	887.5	4.98	4.96
XP4	97.05	96.71	335	335	5.08	5.06
SP8	98.41	97.58	245.5	245	5.01	5.02
SP10	97.79	97.21	350	350	5.02	5.01

## CONCLUSION

From this study, we concluded that by combining PF127 with other in situ gelling- or viscosity-enhancing polymers we could not only able to reduce the concentration of PF127 from 25% to 15% w/v but also able to reduce the individual polymer concentrations, i.e. carbomer and sodium alginate, than if they were used alone as *in situ* gelling polymers. Thus, we could reduce the side effects and increase the patient compliance. Such systems formulated with low buffer capacity and having combination of polymers with in situ gelation by two different mechanisms, can be easily transformed into gel upon instillation into the cul-de-sac of the dye because of rapid temperature change from 25 °C to 37 °C (body temperature) and rapid neutralization of Carbomer by pH of tear fluid and in case of sodium alginate by cation Ca+2 of the tear fluid of the eye.

The overall results of the study indicate that such an *in situ* gelling system is an excellent carrier for prolonged ophthalmic delivery of Cromolyn sodium. The gel formed in situ at lachrymal fluid conditions (37 °C, 7.4 pH, Ca+2) exhibited sustained drug release for more than 10 h. The system is easy to instill into eyes because of its low viscosity and has an excellent ocular tolerance.
